# MEMMAL: A tool for expanding large-scale mechanistic models with machine learned associations and big datasets

**DOI:** 10.3389/fsysb.2023.1099413

**Published:** 2023-03-09

**Authors:** Cemal Erdem, Marc R. Birtwistle

**Affiliations:** 1 Department of Chemical and Biomolecular Engineering, Clemson University, Clemson, SC, United States,; 2 Department of Bioengineering, Clemson University, Clemson, SC, United States

**Keywords:** mechanistic modeling, machine learning, SBML, multi-omics, data integration

## Abstract

Computational models that can explain and predict complex sub-cellular, cellular, and tissue-level drug response mechanisms could speed drug discovery and prioritize patient-specific treatments (i.e., precision medicine). Some models are mechanistic with detailed equations describing known (or supposed) physicochemical processes, while some are statistical or machine learning-based approaches, that explain datasets but have no mechanistic or causal guarantees. These two types of modeling are rarely combined, missing the opportunity to explore possibly causal but data-driven new knowledge while explaining what is already known. Here, we explore combining machine learned associations with mechanistic models to develop computational models that could more fully represent cellular behavior. In this proposed MEMMAL (MEchanistic Modeling with MAchine Learning) framework, machine learning/statistical models built using omics datasets provide predictions for new interactions between genes and proteins where there is physicochemical uncertainty. These interactions are used as a basis for new reactions in mechanistic models. As a test case, we focused on incorporating novel IFNγ/PD-L1 related associations into a large-scale mechanistic model for cell proliferation and death to better recapitulate the recently released NIH LINCS Consortium MCF10A dataset and enable description of the cellular response to checkpoint inhibitor immunotherapies. This work is a template for combining big-data-inferred interactions with mechanistic models, which could be more broadly applicable for building multi-scale precision medicine and whole cell models.

## Introduction

The molecular signaling mechanisms of cancer cells are highly heterogenous, leading to treatment resistance and recurrence. Thus, the need for personalized interventions to block tumor growth is high. The traditional drug discovery pipeline is comprised of extensive trial-and-error experiments, testing thousands of chemicals, refining their structure for safety and toxicity, and administering years of clinical trials. This burden might be reduced by understanding the underlying molecular mechanisms with the help of computational models (Yu et al., 2018; Saez-Rodriguez and Blüthgen, 2020).

Computational tools and models are becoming indispensable in medical research, where a cycle of experimentation and computation is used to learn about and test new hypotheses.

The models guide experimental hypothesis generation, and experimental observations enable fine-tuning computational models to understand the biological phenomena. Owing to the advances in wet-lab experimental techniques and tools, “Big Data” repositories become more prominent each year. The knowledge base of these databases includes genomics, proteomics, epigenomics, and clinical information (Barrett et al., 2012; Uhlen et al., 2015; Subramanian et al., 2017; Hoadley et al., 2018; Wishart et al., 2018; Nusinow et al., 2020). To understand the underlying biological facts, analysis of the wealth of the aforementioned big datasets should become more practical and go beyond context-dependent and scope-limited biological events.

**FIGURE 1:**
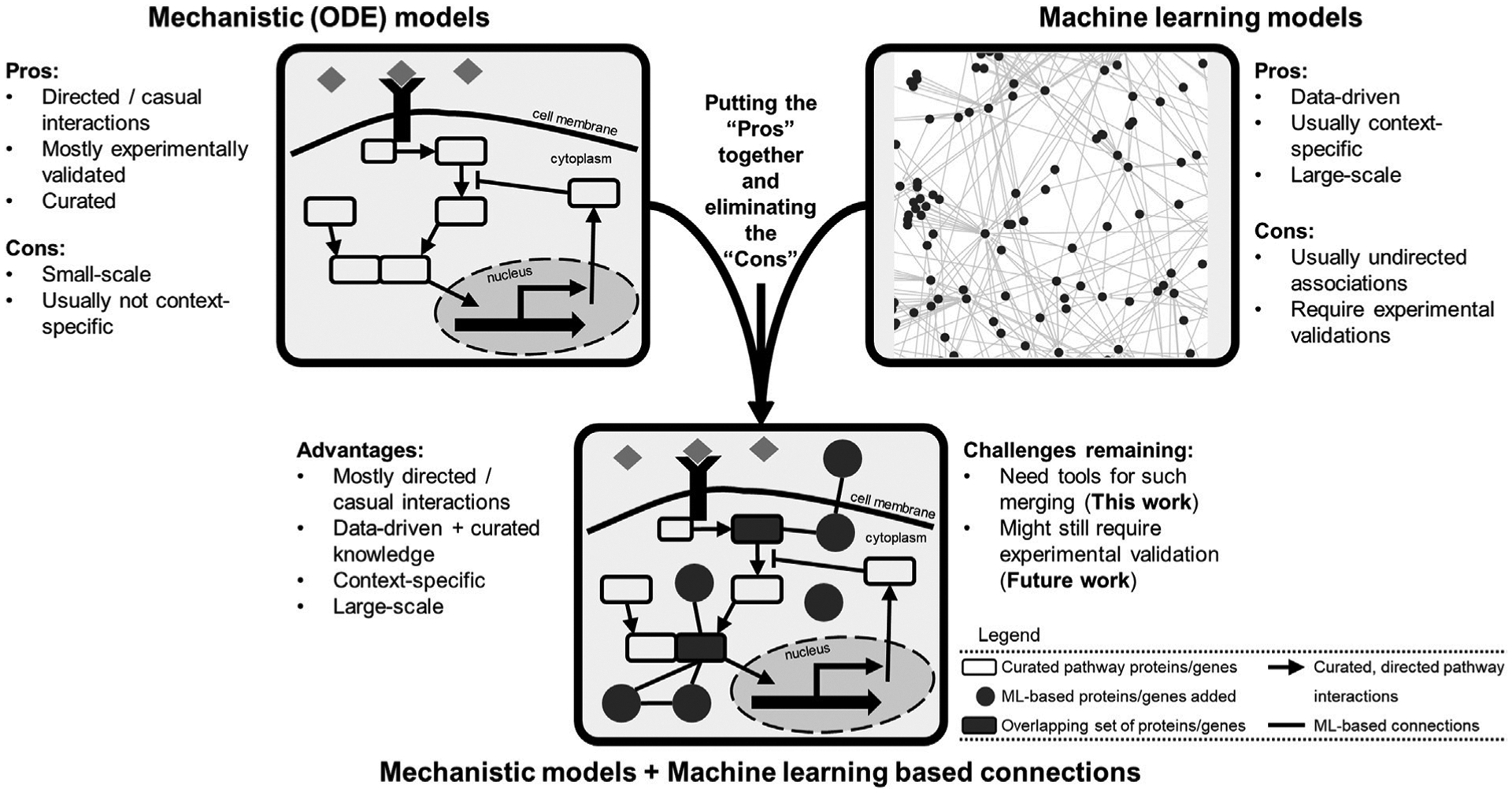
Different computational modeling types of biological data possess a variety of pros and cons and provide an opportunity for model merging. The mechanistic models are mostly curated, usually small-scale, causal networks of signaling pathways. Machine learning models are data-driven, large-scale, and usually correlative associations. Combining these two modes of modeling provides an opportunity for creating larger scale data-informed models to generate novel hypotheses for experimental validation. The merged model would include curated lists of pathway genes (species) as well as genes with new connections inferred *via* machine learning models. The final model structure could represent a collection of overlapping genes (and gene products) and interactions present in both lists.

Building computational models that explain and predict such highly heterogenous and complex cellular responses is no easy task. The popular mechanistic models are sets of detailed equations describing curated knowledge of what is happening within the cells. Such models (Bouhaddou et al., 2018; Fröhlich et al., 2018; Münzner et al., 2019) are usually small in scale: tens of equations and 10s–100s of model species (Figure 1). Another popular class is machine learning based models, which are data-driven, descriptive, and mostly large-scale (genome-wide or exome-wide) (Malta et al., 2018; Wong and Yip, 2018; Yu et al., 2018; Yang et al., 2019). These types of models are generally coined as black-box models because although they perform well in precision/recall metrics, how they do so is blurry (Figure 1). So far in the literature, these two types of models are rarely combined, missing the opportunity to generate new knowledge while explaining what is already known (Baker et al., 2018).

Here, we explore a combination of both methods to develop better models that will more completely represent generated biological knowledge and introduce MEMMAL (MEchanistic Modeling with MAchine Learning) framework. MEMMAL processes connections inferred *via* machine-learning pipelines (i.e., MOBILE (Erdem et al., 2022a)) as new interactions into mechanistic models (i.e., SPARCED (Erdem et al., 2022b)) to better recapitulate available datasets (i.e., the recently-released MCF10A dataset (Gross et al., 2022)). The NIH-LINCS Consortium and MCF10A Common Project recently released this dataset, consisting of multiple omics assay types on breast epithelial MCF10A cell line. MOBILE is a new pipeline to integrate multi-omics datasets and identify context-specific interactions. SPARCED is one of the largest mechanistic models of mammalian cells and is an open-source, human-interpretable, and easy to alter modeling format. Here we focused on incorporating novel IFNγ/PD-L1 related associations into the SPARCED model to enable description of the cellular response to checkpoint inhibitor immunotherapies. This work is a template for combining big data, machine-learning-inferred interactions with mechanistic models, which could be more broadly applicable towards building multi-scale precision medicine and whole cell models.

## Materials and methods

In this work, we use ligand-specific interactions between genes as new connections in a large-scale mechanistic model to study the effect of the newly added gene interactions in model responses. It is important to note that MEMMAL is agnostic to the specific tool used to nominate new associations, and the base mechanistic model used; the below are simply chosen as illustrative.

### MOBILE

MOBILE is a recent tool for finding context-specific network features by integrating pairs of omics datasets (Erdem et al., 2022a). In short, statistical associations are calculated between pairs of chromatin accessibility regions, mRNA expressions, and protein/phosphoprotein levels. Lasso (least absolute shrinkage and selection operator) regression models are run in replicate to select coefficients with high occurrence rates (Tibshirani, 1996; Erdem et al., 2016; Erdem et al., 2022a). The so-called Integrated Association Networks (IANs) are generated by combining the association networks inferred for RPPA (reverse phase protein array)+RNAseq and RNAseq + ATACseq data inputs. Finally, the IANs are coalesced into gene-level networks: nodes representing genes of the assay analytes and edges representing the inferred Lasso coefficients. From MOBILE generated IFNγ-specific IAN, a sub-network of connections between canonical interferon genes, PD-L1, and PD-1 is filtered to obtain a 297 node + 321 edge module. Then, only the interactions with IRF1, PD-L1, PD-1, and STAT1 are retained as input for MEMMAL.

### SPARCED

The starting mechanistic model used in this work is obtained from the SPARCED repository (github.com/birtwistlelab/SPARCED/tree/develop) (Erdem et al., 2022b). It is a recent framework for large-scale mechanistic modeling that enables model file creation using simple text files as input with minimal coding requirements. In short, a set of annotated text files are constructed to define model specifics. Then, Jupyter notebooks are used to process these files and create community-standard model file type called Systems Biology Markup Language (SBML) (Hucka et al., 2003; Keating et al., 2020). The software was first built to replicate the one of the largest mammalian single-cell mechanistic model of proliferation and death signaling (Bouhaddou et al., 2018; Erdem et al., 2022b). Then, an expanded SPARCED model was created to include IFNγ signaling and SOCS1 crosstalk to growth pathways and the new model was named as SPARCED-IFNG-SOCS1 (Erdem et al., 2022b). This final model and its input files are used as the basic model in this work and is modified further with the MOBILE inferred set of new connections.

### MEMMAL

#### Jupyter notebooks

MEMMAL pipeline is composed of multiple Jupyter notebooks defined below and detailed steps given in Supplementary Table S1.

1. enlargeModel notebook: As the core of MEMMAL, this Jupyter notebook processes the machine learning model inferred connections list and creates Species (genes, mRNAs, proteins, phosphoproteins), RateLaws (the reaction format and related parameters), Gene Regulatory Interactions (defining transcriptional activators and repressors) and finds relevant new omics data from LINCS datasets. The input files for SPARCED pipeline are then updated followed by model compilation and simulation steps.
  The pipeline starts by finding the unique list of genes from the MOBILE associations input. Then, for each unique gene added we create species for the active gene, inactive gene, mRNA, and protein (phosphoproteins as well if the gene has corresponding phosphoprotein measurements). The species initial conditions are updated using LINCS (Gross et al., 2022), MCF10A (Bouhaddou et al., 2018), or other literature datasets (Schwanhäusser et al., 2011). The experimental data in molecules per cell (mpc) are converted into nanomolar (nM) concentration and the corresponding values are updated. Next, first-order translation, transcription, and protein and mRNA degradation reactions are created and the rate laws are defined. The rate constants are set using literature data (Schwanhäusser et al., 2011) or set to the mean value of the corresponding reaction parameter values for existing genes in SPARCED. The mRNA and protein degradation rate constants are set using literature half-life data $\left(\text{kTCd}=\frac{\text{log}(2)}{mRNA_{\text{half}-\text{life}}};\text{kTLd}=\frac{\text{log}(2)}{{\text{protein}}_{\text{half}-\text{life}}}\right),$ basal transcription rate constants using the equation ((kTCd*mRNA_*count*_)*(*kG*_*in*_ + *kG*_*ac*_)/(*kG*_ac_*Gene Copy Number) where *kG*_*in*_ and kG_ac_ are rate of gene inactivation and activation, respectively. The translation rate constants are set using the equation (*protein*_concentration_*kTLd/mRNA_*concentration*_).
  Importantly, for this work we specify that all associations are gene regulatory mechanisms, and for each association, two transcriptional regulation connections are created: the protein species of gene1 activates/represses gene2 expression and protein of gene2 activates/represses gene1 expression. That however is because of the specific submodel of interest here being a gene regulatory subnetwork and future implementations would need to be considered case-by-case. These gene regulatory reactions are modeled as Hill equations as defined for other gene regulatory reactions in SPARCED (Erdem et al., 2022b). The Hill equation parameters are: i) n_A_: Hill coefficients set to “4” for all new reactions and ii) K_A_ the concentration for half-maximal transcriptional output effect, initially set to half of the transcriptionally regulating protein concentration. The values of these K_A_ parameters are fitted later, as described below. Finally, the updated input files are written into text files for model creation and compilation.
2. createModel_o4a notebook: The Jupyter notebook to create an integrated SBML version of the SPARCED type models (Erdem et al., 2022b). Creating the model file fully in SBML format provides extensive speed-up of simulations. The newly updated input files by enlargeModel notebook are used to create and compile the expanded model.
3. runModel notebook: This Jupyter notebook is used to simulate and explore multiple scenarios for the new model.
4. enlargeSBMLModel notebook: This Jupyter notebook contains an example to enlarge any SBML model using user defined lists of species, reactions, and parameters. We provide an example use of enlargeModel notebook created lists of model elements to expand the SBML file of IFNγ/JAK/STAT signaling pathway (Yamada et al., 2003).
5. testMEMMAL notebook: This Jupyter notebook contains commands to run MEMMAL from start to finish. It calls the first three notebooks and plots the figure panels.

#### Input files

1. Compartments, GeneReg, OmicsData, RatelawsNoSM, Species, and Initializer text files: SPARCED input files for the SPARCED-IFNG-SOCS1 model from (Erdem et al., 2022b).
2. IRF1_PDL1sub: MOBILE derived associations list from (Erdem et al., 2022a). Steps to obtain the list are given in Supplementary Figure S1.
3. RNAseqDataLINCS: RNAseq data in log2(fpkm+1) format.
4. RPPADataLINCS, RPPADataStdLINCS, and RPPADataStdLINCSfc: Median normalized RPPA data in log2 format. “Std” refers to standard deviation of triplicate measurements. “fc” refers to fold-change with respect to time point zero.
5. Schwanhausser2011: Literature data on mRNA and protein half-lives (Schwanhäusser et al., 2011).
6. Supplementary_Data_22: Transcriptomic and proteomic data for MCF10A cells (Erdem et al., 2022b).

#### Output files and folders

1. GeneReg_MM, OmicsData_MM, RatelawsNoSM_MM, and Species_MM text files: Updated/expanded input files with new connections and data.
2. “Model name.txt” [i.e., MEMMAL_orig.txt]: Model file in Antimony format (Smith et al., 2009).
3. “Model name.xml” [i.e., MEMMAL_orig.xml]: Model file in SBML format (Keating et al., 2020).
4. “Model name folder” [i.e., MEMMAL_orig]: Compiled model folder created by AMICI package (Fröhlich et al., 2020; Weindl et al., 2020).

#### Parameter fitting

The new parameter values were initially set using literature data or existing model parameters. We then estimated some of them in a semi-automated way. First, the basal transcription (mRNA production) rate constants of the new mRNAs species (eight in total) are fitted one at a time, in the order of species added to the model. If the mRNA level was not at steady state, degrading or accumulating in no ligand (growth factors or IFNγ) stimulation simulations, the parameter value is estimated by varying it uniformly (15 points) within three orders of log10-magnitude of the default value. Then, the best-fit value that yields a constant level is manually adjusted for better fit if possible. Finally, such parameter values are kept constant and the next is explored. One of the mRNA degradation parameters (of FAM83D) was also fitted similarly.

The values for the K_A_ (half-maximal) concentrations of the newly added gene regulatory reactions were adjusted using the LINCS mRNA (ACSL5, BST2, CLIC2, FAM83D, HIST2H2AA3, and METAP2) and protein (IRF1 and PD-L1) time course data with EGF and EGF + IFNγ stimulation. The model, starting from an initial steady-state condition in the absence of growth factors (from above), is simulated for 48 h with EGF (1.5625 nM) or EGF (1.5625 nM) + IFNγ (1.1834 nM) treatment. The K_A_ for each new gene regulatory interaction (27 total) is varied uniformly (15 points) within three orders of log10-magnitude of the default value (half the regulating protein species concentration) and both stimulation conditions are simulated. The sum-of-squared errors between simulation and the data is evaluated for each, and the value giving minimum error is chosen. In some cases, the value with minimum error is manually adjusted between originally sampled values to achieve better fit. These fitted K_A_ parameter values are reported in the runModel notebook.

#### Code availability

MEMMAL code is available at the GitHub repository github.com/cerdem12/MEMMAL.

## Results

### Large-scale mechanistic models can become larger and more precise by expansion using machine learned relationships

There are only a handful of large-scale (hundreds of genes, thousands of species) mechanistic signaling pathway models in the literature (Fröhlich et al., 2018). Usually, such big models are constructed by bottom-up modeling or by semi-manual stitching of previously published models (Bouhaddou et al., 2018). Both approaches are time consuming, manually curated, and biased for including/excluding model components: genes, proteins, post-translational modifications, interactions, or even cellular compartments. Here, we tackle this “what-to-add” problem by using association networks inferred *via* data-driven machine learning algorithms.

**FIGURE 2:**
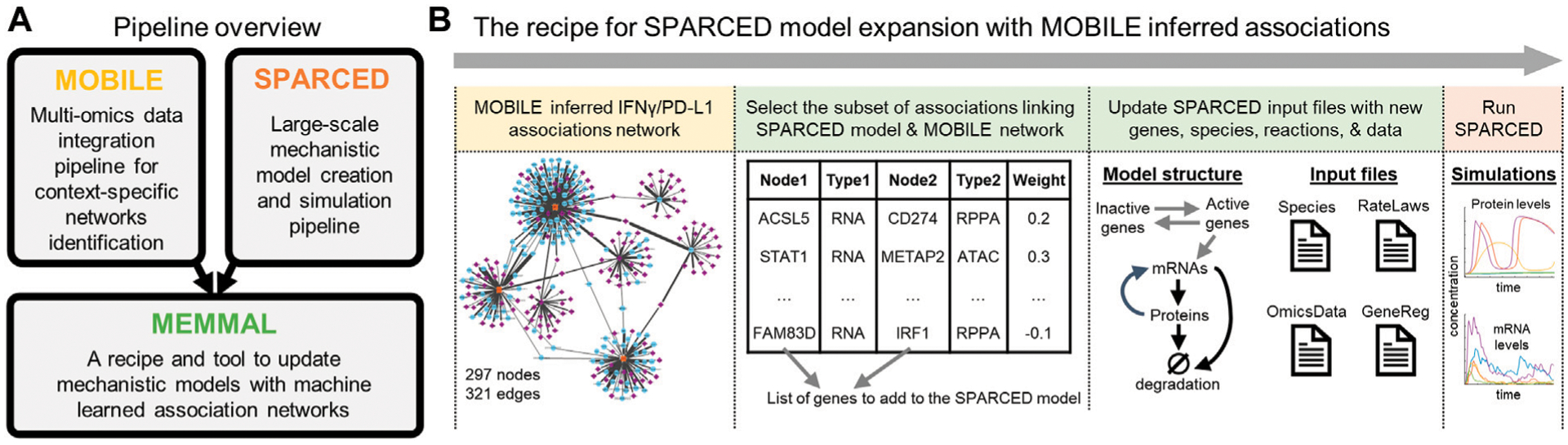
MEMMAL is a pipeline to merge mechanistic modeling with machine learning (A) The MEMMAL pipeline combines mechanistic models created by SPARCED with association networks generated *via* MOBILE pipeline (B) The recipe for MEMMAL pipeline starts by obtaining a set of connections not presented in the candidate mechanistic model. Here, the novel gene-level connections list is inferred *via* the MOBILE tool and then filtered for overlap with SPARCED model genes. Next, this candidate network is imported into SPARCED environment, where the MEMMAL enlargeModel Jupyter notebook processes the network file and updates SPARCED input files The nodes (genes) of the IFNG/PD-L1 subnetwork are used to create new genes and species (mRNAs, proteins, phosphoproteins) for SPARCED. The new genes can get activated/inactivated as described in SPARCED. The expanded MEMMAL model is created and compiled by default SPARCED model notebooks. The final step in MEMMAL is to run user defined exploratory simulations to gain insights on the effects of new connections added.

The Mechanistic Modeling with Machine Learning (MEMMAL) tool presented here (Figure 2) is comprised of scripts to expand mechanistic models created using SPARCED pipeline (Erdem et al., 2022b) with candidate connections generated by the tool called MOBILE, a recent pipeline for multi-omics data integration (Erdem et al., 2022a). However, other tools and models could be used in their place; they are simply used to demonstrate the approach. For now, the MEMMAL Jupyter notebooks process these new connection candidates to update SPARCED input files, taking advantage of their modular structure for model building (github.com/birtwistlelab/SPARCED/tree/develop). Here, we combine novel connections inferred *via* MOBILE with a large-scale mechanistic model called SPARCED to add an immune-checkpoint related sub-module to the existing pan-cancer model to study effects of the newly added gene products on the regulation of Interferon Regulatory Factor 1 (gene name IRF) and Programmed Death Ligand 1 (PD-L1, gene name CD274) upon interferon-gamma (IFNγ, gene name IFNG) stimulation.

### MOBILE pipeline integrated LINCS MCF10A multi-omics dataset to infer ligand-specific associations

The normal-like breast epithelial cell line MCF10A was recently profiled with multiple assay types under multiple ligand stimulation conditions (Gross et al., 2022). Using this newly released multi-omics dataset, our lab introduced the MOBILE pipeline for data integration and showed how ligand-specific associations can be inferred (Erdem et al., 2022a). One of the ligands included in the LINCS study that induced MCF10A growth inhibition was interferon-gamma (Gross et al., 2022). We previously analyzed the LINCS MCF10A dataset to find IFNγ-specific associations that nominate novel connections with the PD-L1 (gene name CD274) axis (Erdem et al., 2022a). IFNγ can induce transient PD-L1 expression, a transmembrane protein that binds to its receptor PD-1 on T-cells (Abiko et al., 2015; Thiem et al., 2019; Ju et al., 2020). This binding inhibits tumor clearance, where targeted therapies towards these proteins are a new class of anti-cancer drugs: the immune checkpoint inhibitors (Gong et al., 2018). However, inter- and intra-tumor variability of PD-L1 expression results in heterogeneous patient responses and makes the response predictions a challenge (Wu et al., 2019). A more thorough understanding of the regulatory mechanism of PD-L1 expression could help inform new immunotherapeutic drugs or treatment options.

Applying MOBILE, we generated a data-driven IFNγ-specific integrated associations network, which had 297 nodes (genes) and 321 edges (connections) (Figure 2B and Supplementary Figure S1). We further filtered this network by looking for connections with STAT1 (the only overlapping gene with the mechanistic model). The final list of candidate connections had nine genes (ACSL5, BST2, CD274, CLIC2, FAM83D, HIST2H2AA3, IRF1, METAP2, and STAT1) and 14 connections. The list is imported into the SPARCED environment to start altering the existing mechanistic model structure (Figure 2B and Supplementary Figure S1).

### SPARCED modeling makes mechanistic model expansions easy

**FIGURE 3:**
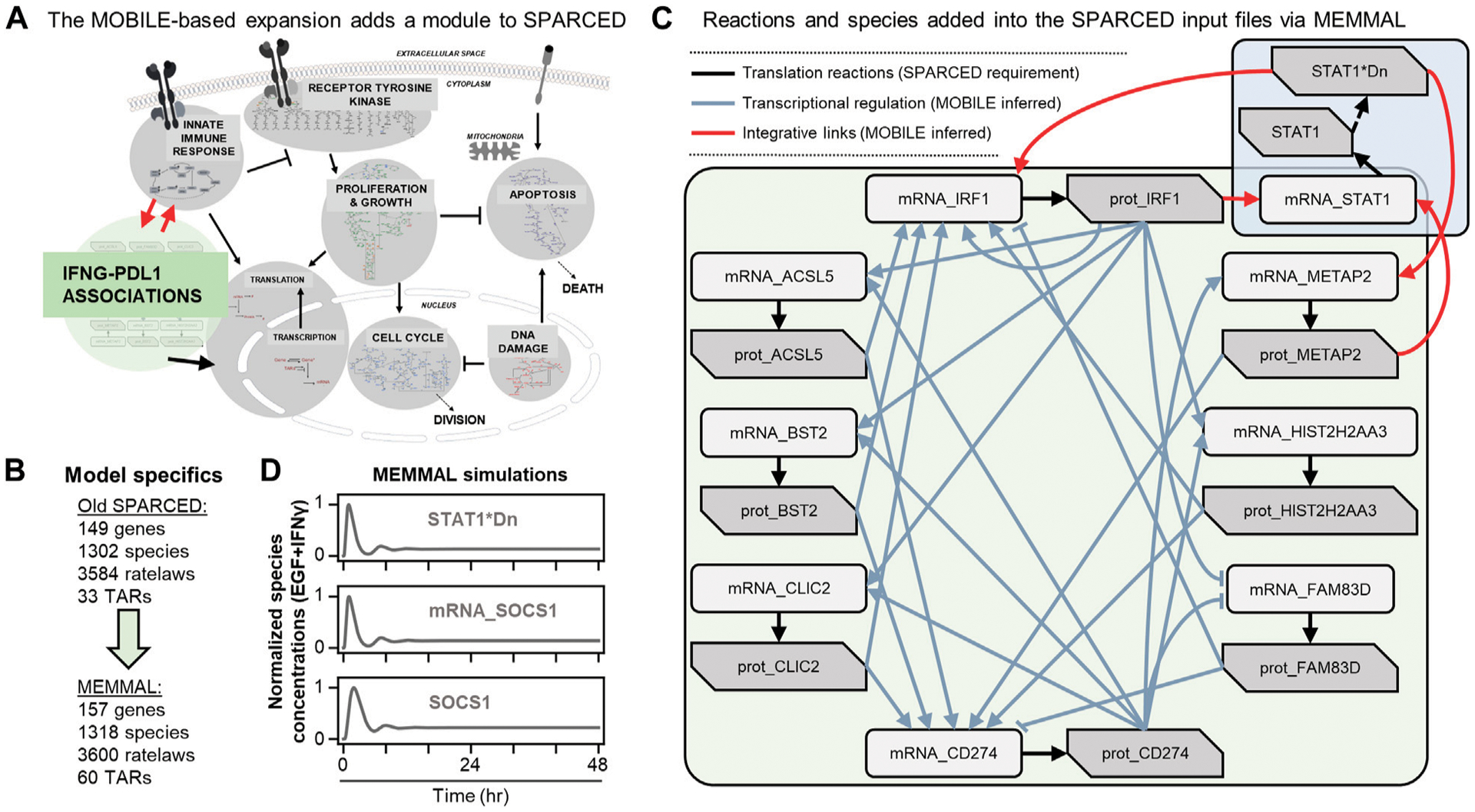
MOBILE inferred IFNγ/PD-L1 network nodes and connections are inserted into the SPARCED-IFNG model using MEMMAL **(A)** The SPARCED network is enlarged to include a sub-network spanning innate immune response and PD-L1 regulation **(B)** The final MEMMAL model is 157 genes, 1,318 species, and 3,600 ratelaws, 60 TARs, and 3,885 parameters **(C)** The reactions added into SPARCED include translation (black arrows). The connections from MOBILE are modeled as transcriptional activation and repression (TAR) reactions in MEMMAL (gray arrows). The TAR reactions linking existing SPARCED species with the newly added species are represented as integrative links (red arrows) **(D)** The final MEMMAL model recapitulates canonical transient STAT1 and SOCS1 activation in response to IFNγ stimulation in MCF10A cells (Erdem et al., 2022b). Normalized simulation trajectories of the activated nuclear STAT1 dimer (STAT1*Dn), SOCS1 mRNA (mRNA_SOCS1), and free SOCS1 protein (SOCS1) are shown (solid gray lines).

SPARCED is a recent software (Erdem et al., 2022b) and modeling framework for large-scale mechanistic modeling. It enables SBML model file creation using simple text files as input with minimal coding requirements. Jupyter notebooks (Kluyver et al., 2016) are used to process the input files and to create the model files. The software was first built to replicate the largest mammalian single-cell mechanistic model of proliferation and death signaling (Bouhaddou et al., 2018) and was then expanded to include a new sub-module of IFNγ signaling (Yamada et al., 2003). So, the starting mechanistic model in this work, SPARCED-IFNGSOCS1 already includes an IFNγ submodule (Figure 3A, gray background), with a total of 149 genes, 1,302 species, and 3,584 ratelaws (Figure 3B).

### MEMMAL incorporates MOBILE-inferred gene-level statistical associations into SPARCED as gene regulatory mechanisms

The list of candidate connections from MOBILE pipeline are processed *via* MEMMAL enlargeModel notebook to add rows and update SPARCED input files (Figure 2B). As a default SPARCED requirement, each gene node from MOBILE list is interpreted to create active gene, inactive gene, mRNA, and protein species, with relevant basic reactions: gene switching, transcription, translation (Figure 3C, black arrows), mRNA degradation, and protein degradation. Importantly, the MOBILE inferred connections are interpreted as transcriptional activator and repressor (TAR) reactions (Figure 3C) because the MOBILE inferred connections are obtained by looking at pairs of mRNA-protein and chromatin region-mRNA dataset pairs. A logical way a protein affecting another mRNA’s expression level is by transcriptional regulation. Additionally, a highly open chromatin region can permit transcription, which potentially yields higher mRNA expression and thus another gene regulatory connection. So, all the candidate associations are treated as TARs in the current MEMMAL pipeline. For future work, users should decide how to handle such connections.

The negative valued associations here are treated as inhibitory whereas the positive magnitude connections are added as activators (Figure 3C, gray and red arrows). Some of the transcriptional activators are labeled as “integrative links” because they connect existing SPARCED model genes with the new gene species (Figure 3C, red arrows). After all the input files are updated, createModel_o4a Jupyter notebook is used to create and compile the new SBML model file (Figure 2B). The MEMMAL expansion of SPARCED *via* MOBILE inferred network resulted in the addition of eight genes, 16 species, 16 signaling reactions, and 27 transcriptional regulatory mechanisms (Figures 3A, B). With the current addition, the SPARCED model now includes an IFNγ-PD-L1 submodule (Figure 3A, green background).

Following model expansion, we first verified the model can recapitulate previous observations (Figure 3D). We show that inclusion of new species and reactions did not alter canonical STAT1-SOCS1 response to IFNγ stimulation. Previous studies have shown that in response to IFNγ, STAT1 and SOCS1 show transient activation over several hours followed by damped oscillations before reaching a steady state slightly higher than the baseline levels (Yamada et al., 2003). In the model, IFNγ treatment leads to transient STAT1 activation by inducing its phosphorylation, dimerization, and translocation to nucleus (Figure 3D, top panel). Nuclear STAT1 dimer acts as an activating transcription factor for SOCS1 and induces SOCS1 mRNA production (Figure 3D, middle panel), which then causes SOCS1 protein levels to increase (Figure 3D, bottom panel). Moreover, as reported previously in (Erdem et al., 2022b), IFNγ does not induce significant changes in MAPK signaling but leads to a slight decrease in early AKT response (Supplementary Figure S2).

### MEMMAL model offers exploration of the effect of novel connector genes on the expression of PD-L1 expression in response to IFNγ

**FIGURE 4:**
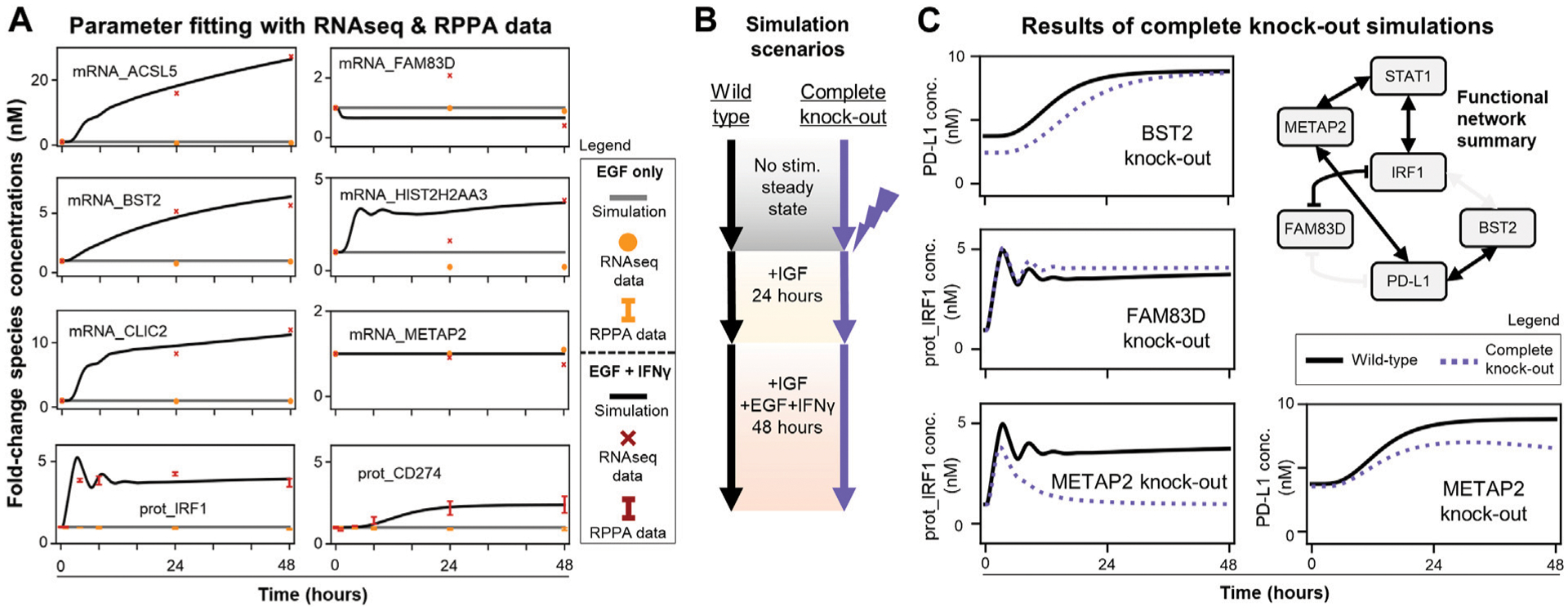
MEMMAL can replicate the previous SPARCED-IFNG model and offers new insights into IFNγ regulation of IRF1 and PD-L1 dynamics **(A)** MEMMAL model parameters are fitted to recapitulate experimental data from LINCS RNAseq and RPPA assays. Fold-changes are shown for data (dots, crosses, and error bars, STD) and simulations (solid lines). Most mRNAs and IRF1 and PD-L1 (gene name CD274) are induced by IFNγ **(B)** Simulation scenarios to test the effects of newly added genes. The parameter fitted MEMMAL model is simulated with reported perturbation under IGF1 stimulation (basal growth condition) and then stimulated with additional EGF + IFNγ for 48 h **(C)** Comparison of complete gene knock-out perturbation scenario (dotted lines) to wild-type (no perturbation, black lines) condition shows genes with induced IRF1 and PD-L1 changes. Among the newly added genes, METAP2 induces the greatest change: a complete recession of IRF1 response and decreased PD-L1 steady-state level. The network diagram (summary of Figure 3C) shows the connections among functional genes and STAT1, with non-functional edges faded out.

Since the modified model passed these quality control checks, the next step was to fit new unknown parameters to recapitulate experimental time-course data for newly added genes (RNAseq: ACSL5, BST2, CLIC2, FAM83D, HIST2H2AA3, METAP2 and RPPA: IRF1, PD-L1) (Figure 4A). These 27 + 16 (43 total) unknown parameters were the half-maximal concentrations for the Hill functions underlying the new gene regulatory reactions and protein/mRNA degradation rate constants. The data show IFNγ induces transcription of ACSL5, BST2, CLIC2, and HIST2H2AA3 and expression of both IRF1 and PD-L1 with no sustained induction of FAM83D and METAP2, and the fitted model captures these trends. There are only two discrepancies where the model could not capture: 24-h time point data of FAM83D and HIST2H2AA3 mRNA levels. However, the model can recapitulate the increasing trend of mRNA_HIST2H2AA3 and fit the last time points for both species levels. The runModel Jupyter notebook reports the final updated parameter values and scripts to compare simulation trajectories with LINCS data (Figure 4A).

After acceptable agreement was achieved between simulations and experimental mRNA and protein levels (Figure 4A), we simulated scenarios (Figure 4B) to explore the effects of new genes on the IRF1 and PD-L1 responses. We wanted to nominate the new connections predicted to be most important in regulating PD-L1 expression. To do this we compared wild-type simulations (new model with fit parameters) to single gene knock-out simulations (protein, gene, and mRNA levels set to zero) (Figures 4B,C).

Only BST2, FAM83D, and METAP2 knock-outs had observable effects on simulated PD-L1 and/or IRF1 dynamics (Figure 4C). Knocking out other newly added genes (ACSL5, CLIC2, HIST2H2AA3) had no significant effects and thus are not shown here. Perturbing BST2 caused a small decrease in initial PD-L1 levels, which later reaches to wild-type response levels (Figure 4C, top row). Perturbing FAM83D only slightly increased steady-state IRF1 levels (Figure 4C, middle row). Perturbing METAP2 caused a significant decrease in late IRF1 and PD-L1 responses (Figure 4C, bottom row). We summarized all these knock-out response observations with the candidate gene regulatory network in Figure 3C to show a functional network with possibly causal links only (Figure 4C). These results demonstrate that mechanistic models with machine learning derived connections can nominate genes for follow-up experimental studies.

## Discussion

Combining and synergizing machine learning with mechanistic modeling could bring clinically predictive computational models and personalized medicine closer to reality. To that end, here we introduced a recipe to expand a large-scale mechanistic model with machine learned connections between gene products. Because understanding PD-L1 regulation mechanisms would help us design better therapeutic interventions, we focused on exploring the IFNγ/PD-L1 axis. We used the LINCS MCF10A dataset and added the recently inferred (*via* MOBILE pipeline) IFNγ/PD-L1 connections to the existing SPARCED mechanistic model. We then were able to study the effects of new gene regulatory mechanisms. We showed that perturbing BST2, FAM83D, or METAP2 induces changes in PD-L1 and IRF1 dynamics.

MEMMAL could serve as an initial step towards combining mechanistic models with machine learnt potential connections by providing a rationale for such a merging protocol. MEMMAL protocol first creates genes and gene products (mRNA and protein) if MOBILE list nodes are not already present in SPARCED. It then updates -omics level information for the new genes and adds corresponding reactions. It also assigns transcriptional activator and repressors (based on MOBILE association coefficient sign) and related rate constant parameters. The updated SPARCED input files are then processed *via* modified default Jupyter notebooks to execute desired simulations. The current state of the MEMMAL assumes an overlap (genes) between the mechanistic model and machine learned associations. Although this is not a hard assumption, it also makes logical sense that the effects of added interactions can be explored *via* crosstalk mechanisms.

Although MEMMAL makes use of recent tools from our lab, the idea is applicable to other tools available in the literature. For instance, rule-based modeling software like BioNetGen (Harris et al., 2016) and PySB (Lopez et al., 2013) can also be used for mechanistic model creation and update if machine learning predicted associations are converted into new rules. Another possible application can include INDRA (Gyori et al., 2017) if the new connections are put into suitable sentence format. Such options will be valuable to expand the MEMMAL idea and its applications.

MEMMAL is agnostic to the approach or tool used to identify connections and to the base mechanistic model for expansion. MEMMAL can generate mechanistic ODE models by integrating connections inferred using MOBILE, databases, correlation studies (Lin et al., 2013; Min et al., 2021), kernel-based methods (Mariette and Villa-Vialaneix, 2018; Yang et al., 2018), other machine learning tools (Park et al., 2015; Zhang et al., 2018; Hulot et al., 2021), or direct experiments. For the base model any mechanistic model that can be modified programmatically could be used. To facilitate the use of other models, we have provided a Jupyter notebook (enlargeSBMLmodel) to expand any SBML model with MEMMAL generated lists of new species, reactions, and parameters.

The MOBILE pipeline was used to infer ligand-specific and statistically robust association networks (Erdem et al., 2022a). Here we used a filtered list of connections for interferon-gamma signaling and among them some genes were already shown to be associated with immunotherapeutic signatures including BST2, CLIC2, and FAM83D (Wang et al., 2013; Walian et al., 2016; Xu et al., 2020; Zhou et al., 2020; Mei et al., 2021). In short, BST2 is part of an anti-CTLA4 response in melanoma (Mei et al., 2021) and CLIC2 is a favorable prognosis biomarker (Xu et al., 2020). FAM83D functions in cell growth regulation and is a prognostic marker for multiple cancer types (Wang et al., 2013; Walian et al., 2016). In addition to such pieces of literature support, we can take a step further to explore their mechanistic functionalities by combining these genes and their predicted connections as new interactions in a computational model.

The investigation of the effects of new genes (*via* knock-out simulations) was carried out after fitting the new reaction parameter values to match experimental time course data. The simple semi-automated fitting procedure in this work resulted in a set of parameter values, reported in runModel notebook, but their identifiability is not guaranteed. Because the effects of single gene knock-outs simulations are dependent on such values, a more extensive parameter exploration would build confidence in the predictions of which genes are more important for PD-L1 regulation. Indeed, the AMICI package (Fröhlich et al., 2020) used by SPARCED enables users to do such high-level parameter estimation studies.

In conclusion, the MEMMAL pipeline provides a starting point for merging large-scale mechanistic models with big-data based association networks. We used MEMMAL to test novel candidate interactions for their effect on regulating IRF1 and PD-L1 expression and found that METAP2 is a good candidate yet to be studied experimentally. We believe combining big data, machine learning, and mechanistic models is a valuable direction to unravel novel context-specific mechanisms.

## Supplementary Material

Supplementary Files (zip)

## Data Availability

The original contributions presented in the study are included in the article/Supplementary Material, further inquiries can be directed to the corresponding authors. All the data used in this study are available within the MOBILE repository and adapted from (Gross et al., 2022; Erdem et al., 2022b).

## References

[R1] Abiko K, Matsumura N, Hamanishi J, Horikawa N, Murakami R, Yamaguchi K, (2015). IFN-γ from lymphocytes induces PD-L1 expression and promotes progression of ovarian cancer. Br. J. Cancer 112 (9), 1501–1509. doi:10.1038/bjc.2015.101 25867264 PMC4453666

[R2] Baker RE, Peña JM, Jayamohan J, and Jérusalem A (2018). Mechanistic models versus machine learning, a fight worth fighting for the biological community? Biol. Lett 14 (5), 20170660. doi:10.1098/rsbl.2017.0660 29769297 PMC6012710

[R3] Barrett T, Wilhite SE, Ledoux P, Evangelista C, Kim IF, Tomashevsky M, (2012). NCBI geo: Archive for functional genomics data sets—update. Nucleic Acids Res. 41 (1), D991–D995. doi:10.1093/nar/gks1193 23193258 PMC3531084

[R4] Bouhaddou M, Barrette AM, Stern AD, Koch RJ, DiStefano MS, Riesel EA, (2018). A mechanistic pan-cancer pathway model informed by multi-omics data interprets stochastic cell fate responses to drugs and mitogens. PLoS Comput. Biol 14 (3), e1005985. doi:10.1371/journal.pcbi.1005985 29579036 PMC5886578

[R5] Erdem C, Gross SM, Heiser LM, and Birtwistle MR Multi-Omics Binary Integration via Lasso Ensembles (MOBILE) for identification of context-specific networks and new regulatory mechanisms. bioRxiv. 2022.

[R6] Erdem C, Mutsuddy A, Bensman EM, Dodd WB, Saint-Antoine MM, Bouhaddou M, (2022). A scalable, open-source implementation of a large-scale mechanistic model for single cell proliferation and death signaling. Nat. Commun 13 (1), 3555–3618. doi:10.1038/s41467-022-31138-1 35729113 PMC9213456

[R7] Erdem C, Nagle AM, Casa AJ, Litzenburger BC, Wangfen Y, Taylor DL, (2016). Proteomic screening and Lasso regression reveal differential signaling in insulin and insulin-like growth factor I (IGF1) pathways. Mol. Cell. Proteomics 15 (9), 3045–3057. doi:10.1074/mcp.M115.057729 27364358 PMC5013316

[R8] Fröhlich F, Kessler T, Weindl D, Shadrin A, Schmiester L, Hache H, (2018). Efficient parameter estimation enables the prediction of drug response using a mechanistic pan-cancer pathway model. Cell Syst. 7 (6), 567–579.e6. doi:10.1016/j.cels.2018.10.013 30503647

[R9] Fröhlich F, Weindl D, Schälte Y, Pathirana D, Paszkowski Ł, Lines GT, (2020). Amici: High-performance sensitivity analysis for large ordinary differential equation models. arXiv:201209122 [q-bio] [Internet] Available from: http://arxiv.org/abs/2012.09122. 10.1093/bioinformatics/btab227 PMC8545331 33821950

[R10] Gong J, Chehrazi-Raffle A, Reddi S, and Salgia R (2018). Development of PD-1 and PD-L1 inhibitors as a form of cancer immunotherapy: A comprehensive review of registration trials and future considerations. J. Immunother. cancer 6 (1), 8. doi:10.1186/s40425-018-0316-z 29357948 PMC5778665

[R11] Gross SM, Dane MA, Smith RL, Devlin KL, McLean IC, Derrick DS, (2022). A multi-omic analysis of MCF10A cells provides a resource for integrative assessment of ligand-mediated molecular and phenotypic responses. Commun. Biol 5 (1), 1066. doi:10.1038/s42003-022-03975-9 36207580 PMC9546880

[R12] Gyori BM, Bachman JA, Subramanian K, Muhlich JL, Galescu L, and Sorger PK (2017). From word models to executable models of signaling networks using automated assembly. Mol. Syst. Biol 13 (11), 954. doi:10.15252/msb.20177651 29175850 PMC5731347

[R13] Harris LA, Hogg JS, Tapia JJ, Sekar JA, Gupta S, Korsunsky I, (2016). BioNetGen 2.2: Advances in rule-based modeling. Bioinformatics 32, 3366–3368. doi:10.1093/bioinformatics/btw469 27402907 PMC5079481

[R14] Hoadley KA, Yau C, Hinoue T, Wolf DM, Lazar AJ, Drill E, (2018). Cell-of-Origin patterns dominate the molecular classification of 10,000 tumors from 33 types of cancer. Cell 173 (2), 291–304.e6. doi:10.1016/j.cell.2018.03.022 29625048 PMC5957518

[R15] Hucka M, Finney A, Sauro HM, Bolouri H, Doyle JC, Kitano H, (2003). The systems biology markup language (SBML): A medium for representation and exchange of biochemical network models. Bioinformatics 19, 524–531. doi:10.1093/bioinformatics/btg015 12611808

[R16] Hulot A, Laloë D, and Jaffrézic F (2021). A unified framework for the integration of multiple hierarchical clusterings or networks from multi-source data. BMC Bioinforma. 22 (1), 392. doi:10.1186/s12859-021-04303-4 PMC8336092 34348641

[R17] Ju X, Zhang H, Zhou Z, and Wang Q (2020). Regulation of PD-L1 expression in cancer and clinical implications in immunotherapy. Am. J. Cancer Res 10 (1), 1–11. 32064150 PMC7017746

[R18] Keating SM, Waltemath D, König M, Zhang F, Dräger A, Chaouiya C, (2020). SBML level 3: An extensible format for the exchange and reuse of biological models. Mol. Syst. Biol 16 (8). doi:10.15252/msb.20199110 PMC8411907 32845085

[R19] Kluyver T, Ragan-Kelley B, Pérez F, Granger B, Bussonnier M, Frederic J, (2016). “Jupyter notebooks – A publishing format for reproducible computational workflows,” in Positioning and power in academic publishing: Players, agents and agendas. Editors Loizides F and Schmidt B (Amsterdam: IOS Press), 87–90.

[R20] Lin D, Zhang J, Li J, Calhoun VD, Deng HW, and Wang YP (2013). Group sparse canonical correlation analysis for genomic data integration. BMC Bioinforma. 14 (1), 245. doi:10.1186/1471-2105-14-245 PMC3751310 23937249

[R21] Lopez CF, Muhlich JL, Bachman JA, and Sorger PK (2013). Programming biological models in Python using PySB. Mol. Syst. Biol 9, 646. doi:10.1038/msb.2013.1 23423320 PMC3588907

[R22] Malta TM, Sokolov A, Gentles AJ, Burzykowski T, Poisson L, Weinstein JN, (2018). Machine learning identifies stemness features associated with oncogenic dedifferentiation. Cell 173 (2), 338–354.e15. doi:10.1016/j.cell.2018.03.034 29625051 PMC5902191

[R23] Mariette J, and Villa-Vialaneix N (2018). Unsupervised multiple kernel learning for heterogeneous data integration. Bioinformatics 34 (6), 1009–1015. doi:10.1093/bioinformatics/btx682 29077792

[R24] Mei Y, Chen MJM, Liang H, and Ma L (2021). A four-gene signature predicts survival and anti-CTLA4 immunotherapeutic responses based on immune classification of melanoma. Commun. Biol 4 (1), 383. doi:10.1038/s42003-021-01911-x 33753855 PMC7985195

[R25] Min W, Chang TH, Zhang S, and Wan X (2021). Tscca: A tensor sparse cca method for detecting microRNA-gene patterns from multiple cancers. PLoS Comput. Biol 17 (6), e1009044. doi:10.1371/journal.pcbi.1009044 34061840 PMC8195367

[R26] Münzner U, Klipp E, and Krantz M (2019). A comprehensive, mechanistically detailed, and executable model of the cell division cycle in *Saccharomyces cerevisiae*. Nat. Commun 10 (1), 1308. doi:10.1038/s41467-019-08903-w 30899000 PMC6428898

[R27] Nusinow DP, Szpyt J, Ghandi M, Rose CM, McDonald ER, Kalocsay M, (2020). Quantitative proteomics of the cancer cell line encyclopedia. Cell 180 (2), 387–402.e16. doi:10.1016/j.cell.2019.12.023 31978347 PMC7339254

[R28] Park CY, Krishnan A, Zhu Q, Wong AK, Lee YS, and Troyanskaya OG (2015). Tissue-aware data integration approach for the inference of pathway interactions in metazoan organisms. Bioinformatics 31 (7), 1093–1101. doi:10.1093/bioinformatics/btu786 25431329 PMC4804827

[R29] Saez-Rodriguez J, and Blüthgen N (2020). Personalized signaling models for personalized treatments. Mol. Syst. Biol 16 (1). doi:10.15252/msb.20199042 PMC6951359 32129942

[R30] Schwanhäusser B, Busse D, Li N, Dittmar G, Schuchhardt J, Wolf J, (2011). Global quantification of mammalian gene expression control. Nature 473 (7347), 337–342. doi:10.1038/nature10098 21593866

[R31] Smith LP, Bergmann FT, Chandran D, and Sauro HM (2009). Antimony: A modular model definition language. Bioinformatics 25 (18), 2452–2454. doi:10.1093/bioinformatics/btp401 19578039 PMC2735663

[R32] Subramanian A, Narayan R, Corsello SM, Peck DD, Natoli TE, Lu X, (2017). A next generation connectivity map: L1000 platform and the first 1,000,000 profiles. Cell 171 (6), 1437–1452.e17. doi:10.1016/j.cell.2017.10.049 29195078 PMC5990023

[R33] Thiem A, Hesbacher S, Kneitz H, di Primio T, Heppt MV, Hermanns HM, (2019). IFN-gamma-induced PD-L1 expression in melanoma depends on p53 expression. J. Exp. Clin. Cancer Res 38 (1), 397. doi:10.1186/s13046-019-1403-9 31506076 PMC6737652

[R34] Tibshirani R (1996). Regression shrinkage and selection via the Lasso. J. R. Stat. Soc. Ser. B-Methodological 58, 267–288. doi:10.1111/j.2517-6161.1996.tb02080.x

[R35] Uhlen M, Fagerberg L, Hallstrom BM, Lindskog C, Oksvold P, Mardinoglu A, (2015). Tissue-based map of the human proteome. Science 347 (6220), 1260419. doi:10.1126/science.1260419 25613900

[R36] Walian PJ, Hang B, and Mao JH (2016). Prognostic significance of FAM83D gene expression across human cancer types. Oncotarget 7 (3), 3332–3340. doi:10.18632/oncotarget.6620 26678035 PMC4823109

[R37] Wang Z, Liu Y, Zhang P, Zhang W, Wang W, Curr K, (2013). FAM83D promotes cell proliferation and motility by downregulating tumor suppressor gene FBXW7. Oncotarget 4 (12), 2476–2486. doi:10.18632/oncotarget.1581 24344117 PMC3926842

[R38] Weindl D, Fröhlich F, Stapor P, and Schälte Y (2020). ICB-DCM/AMICI: AMICI v0.11.2. Zenodo[cited 2020 Jul 27] Available from: https://zenodo.org/record/3949231.

[R39] Wishart DS, Feunang YD, Guo AC, Lo EJ, Marcu A, Grant JR, (2018). DrugBank 5.0: A major update to the DrugBank database for 2018. Nucleic Acids Res. 46 (D1), D1074–D1082. doi:10.1093/nar/gkx1037 29126136 PMC5753335

[R40] Wong D, and Yip S (2018). Machine learning classifies cancer. Nature 555 (7697), 446–447. doi:10.1038/d41586-018-02881-7 32034348

[R41] Wu Y, Chen W, Xu ZP, and Gu W (2019). PD-L1 distribution and perspective for cancer immunotherapy—blockade, knockdown, or inhibition. Front. Immunol 10, 2022. doi:10.3389/fimmu.2019.02022 31507611 PMC6718566

[R42] Xu T, Wang Z, Dong M, Wu D, Liao S, and Li X (2020). Chloride intracellular channel protein 2: Prognostic marker and correlation with PD-1/PD-L1 in breast cancer. Aging 12 (17), 17305–17327. doi:10.18632/aging.103712 32915772 PMC7521498

[R43] Yamada S, Shiono S, Joo A, and Yoshimura A (2003). Control mechanism of JAK/STAT signal transduction pathway. FEBS Lett. 534 (1–3), 190–196. doi:10.1016/s0014-5793(02)03842-5 12527385

[R44] Yang H, Cao H, He T, Wang T, and Cui Y (2018). Multilevel heterogeneous omics data integration with kernel fusion. Briefings Bioinforma. 2018, bby115. doi:10.1093/bib/bby115 30496340

[R45] Yang JH, Wright SN, Hamblin M, McCloskey D, Alcantar MA, Schrübbers L, (2019). A white-box machine learning approach for revealing antibiotic mechanisms of action. Cell 177 (6), 1649–1661.e9. doi:10.1016/j.cell.2019.04.016 31080069 PMC6545570

[R46] Yu MK, Ma J, Fisher J, Kreisberg JF, Raphael BJ, and Ideker T (2018). Visible machine learning for biomedicine. Cell 173 (7), 1562–1565. doi:10.1016/j.cell.2018.05.056 29906441 PMC6483071

[R47] Zhang L, Lv C, Jin Y, Cheng G, Fu Y, Yuan D, (2018). Deep learning-based multi-omics data integration reveals two prognostic subtypes in high-risk neuroblastoma. Front. Genet 9, 477. doi:10.3389/fgene.2018.00477 30405689 PMC6201709

[R48] Zhou F, Wang X, Liu F, Meng Q, and Yu Y (2020). FAM83A drives PD-L1 expression via ERK signaling and FAM83A/PD-L1 co-expression correlates with poor prognosis in lung adenocarcinoma. *Int*. J. Clin. Oncol 25 (9), 1612–1623. doi:10.1007/s10147-020-01696-9 32430734

