# Supplementary figures and images for: MEMMAL: A tool for expanding large-scale mechanistic models with machine learned associations and big datasets
Source: Front Syst Biol. Author manuscript; Available in PMC 2024 Jan 24. (PMC10807051; doi:10.3389/fsysb.2023.1099413)

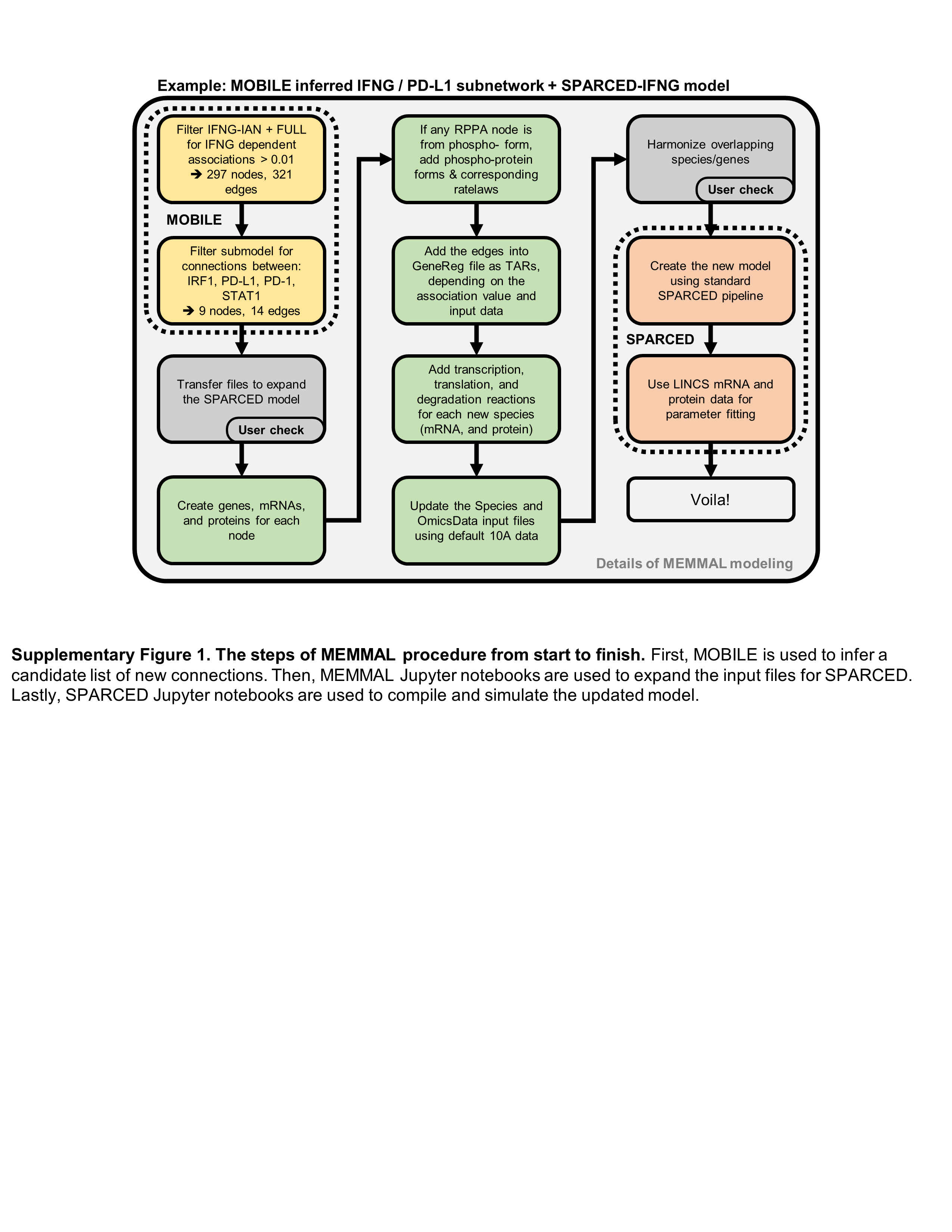

Supplement: Supplementary Files (zip) [file NIHMS1959801-supplement-Supplementary_Files__zip_.zip › Image1_MEMMAL_ A tool for expanding large-scale mechanistic models with machine learned associations and big datasets.TIF]

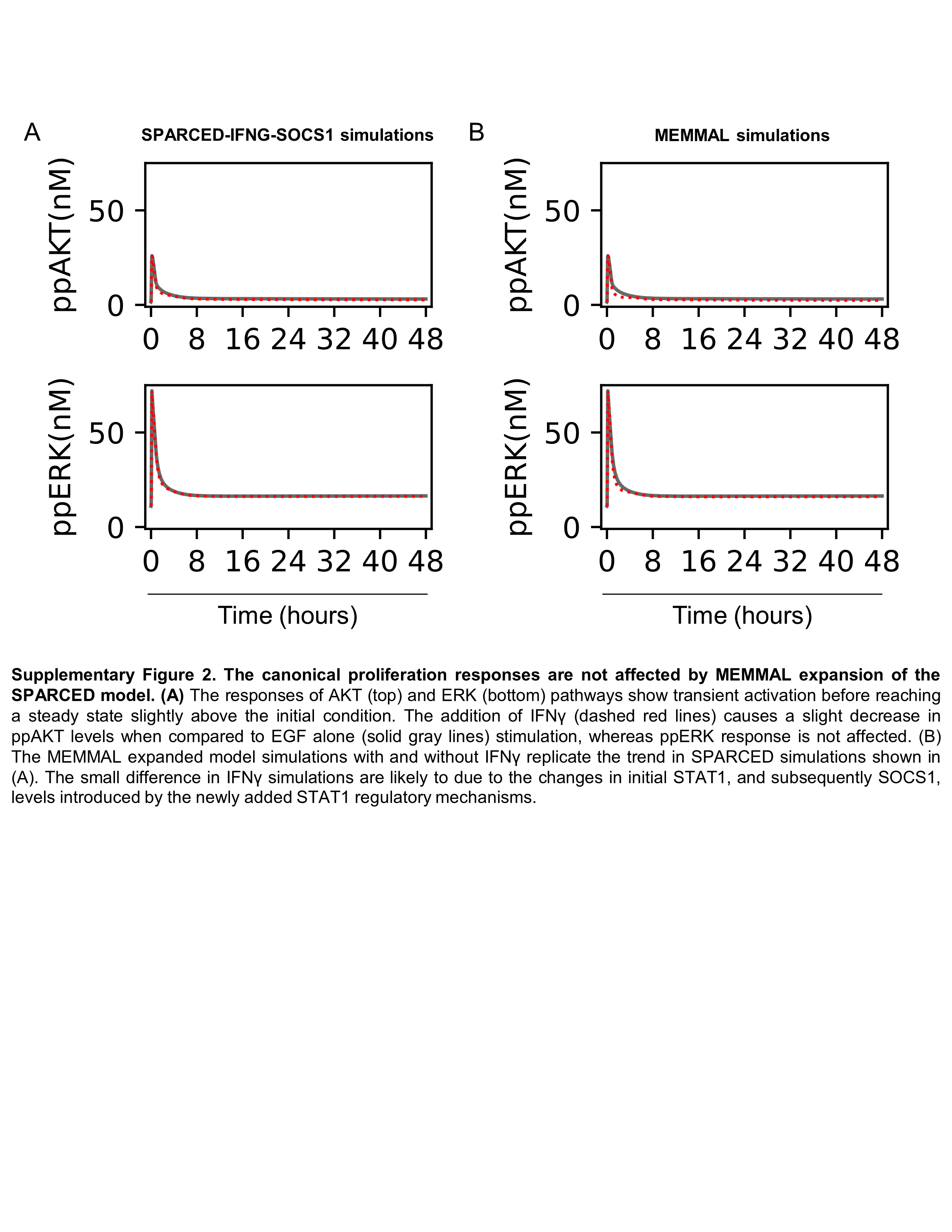

Supplement: Supplementary Files (zip) [file NIHMS1959801-supplement-Supplementary_Files__zip_.zip › Image2_MEMMAL_ A tool for expanding large-scale mechanistic models with machine learned associations and big datasets.TIF]
